# Biomarker‐Based Prediction of OATP1B1 Activity in Clinical Routine—Investigating Coproporphyrins as Markers for Drug–Drug–Gene Interactions

**DOI:** 10.1002/cpt.70335

**Published:** 2026-05-18

**Authors:** Leila Potzel, Anina Zumtaugwald, Anna Bollinger, Chiara Jeiziner, Kurt E. Hersberger, Florine Wiss, Markus L. Lampert, Samuel S. Allemann, Henriette E. Meyer zu Schwabedissen, Céline K. Stäuble

**Affiliations:** ^1^ Biopharmacy, Department of Pharmaceutical Sciences University of Basel Basel Switzerland; ^2^ Pharmaceutical Care Research Group, Department of Pharmaceutical Sciences University of Basel Basel Switzerland; ^3^ Institute of Hospital Pharmacy Solothurner Spitäler AG Olten Switzerland; ^4^ Institute of Hospital Pharmacy Stadtspital Zurich Zurich Switzerland

## Abstract

Coproporphyrin I (CPI) is an established endogenous biomarker for detecting drug–drug interactions (DDIs) involving the hepatic uptake transporter Organic Anion Transporting Polypeptide 1B1 (OATP1B1, gene *SLCO1B1*). While CPI has been extensively studied in healthy volunteers using controlled pre‐ and post‐OATP1B1‐inhibitor sampling, its applicability in samples obtained in clinical routine from multimorbid, polymedicated patients remains poorly characterized. In this study, we evaluated whether single timepoint CPI concentrations obtained in clinical routine can be used to assess *in vivo* OATP1B1 activity by integrating genetic and pharmacological information. Serum CP levels were measured in patients undergoing pharmacist‐led medication reviews following therapy failure or adverse drug reactions. CPI concentrations were analyzed in relation to genotype‐predicted OATP1B1 phenotypes and exposure to drugs with potential impact on OATP1B1, which were identified following a previously published work by Stäuble *et al*., and their interaction with OATP1B1 was further evaluated with the herein presented *in vitro* transporter assay. CPI levels showed an association with drug intake and genotype‐predicted phenotypes. Moreover, patients with decreased‐ or normal function phenotypes taking putative OATP1B1 inhibitors exhibited CPI levels comparable to patients carrying the poor‐function or decreased‐function phenotype without intake of such drugs, respectively, consistent with phenoconversion. These findings provide initial evidence that CPI may serve as an endogenous biomarker for identifying drug–drug‐gene interactions in clinical routine to support a safer and more effective drug therapy. Furthermore, our data emphasize the need for further investigations in larger and more diverse patient populations to validate the findings on CPI.

**Study Highlights**

**WHAT IS THE CURRENT KNOWLEDGE ON THE TOPIC?**

OATP1B1 is a key determinant of the pharmacokinetics of its substrate drugs. Its activity can be altered by function‐impairing polymorphisms in the *SLCO1B1* gene as well as by the administration of medication with OATP1B1 inhibitory potential. Circulating coproporphyrin I (CPI) has been established as a sensitive endogenous biomarker capable of detecting OATP1B1‐mediated drug–drug interactions, and its responsiveness to function‐impairing *SLCO1B1* polymorphisms has likewise been demonstrated. However, current evidence supporting the utility and reliability of CPI has been generated exclusively in controlled clinical studies involving healthy adult volunteers while its applicability in clinical routine so far has not been addressed.

**WHAT QUESTION DID THIS STUDY ADDRESS?**

This study investigates whether CPI could serve as an endogenous biomarker in real‐world clinical samples, drawn at random timepoints from multimorbid, polymedicated patients, to detect drug–drug−gene interactions.

**WHAT DOES THIS STUDY ADD TO OUR KNOWLEDGE?**

This study provides first insights into the application of CPI as a biomarker in real‐world clinical routine samples, rather than only in controlled clinical trials, demonstrating its potential to detect OATP1B1‐related drug–drug–gene interactions in multimorbid, polymedicated patients.

**HOW MIGHT THIS CHANGE CLINICAL PHARMACOLOGY OR TRANSLATIONAL SCIENCES?**

These findings suggest that CPI may have potential utility in real‐world clinical settings for identifying alterations in OATP1B1 activity. While additional studies in larger and more diverse patient cohorts are needed, incorporating CPI measurements into routine workflows could eventually support a more refined assessment of OATP1B1‐related drug–drug–gene interactions.

Personalizing drug therapy remains a major challenge, as multiple factors contribute to interindividual variability in drug response.^1^, ^2^ A major source of variability is pharmacokinetics, governed by molecular mechanisms controlling drug absorption, distribution, metabolism, and elimination (ADME). One key mechanism affecting pharmacokinetics and thereby drug efficacy and safety is the transmembrane transport mediated by drug transporters.^3^ Among these is the hepatic uptake transporter Organic Anion Transporting Polypeptide 1B1 (OATP1B1), encoded by the *SLCO1B1* gene.^4^ OATP1B1 is highly expressed at the sinusoidal membrane of hepatocytes and it facilitates the uptake of various drugs, along with endogenous substrates, from the blood into hepatocytes,^5^ thereby affecting hepatic disposition of its substrates. It is well established that frequently occurring function‐impairing polymorphisms in the *SLCO1B1* gene are associated with elevated systemic concentrations of substrate drugs, which may in turn increase the risk of extrahepatic drug effects.^6^, ^7^ This relationship is particularly well established for statins, where reduced function alleles result in higher drug exposure and a greater likelihood of developing statin‐associated musculoskeletal symptoms (SAMS).^8^ In accordance with current evidence, pharmacogenetic (PGx) guidelines, such as those developed by the Clinical Pharmacogenetics Implementation Consortium (CPIC), recommend genotype‐guided statin therapy to mitigate the risk of adverse drug reactions (ADRs).^8^ Beyond genetic variability, the activity of the hepatic transporter OATP1B1 can also be influenced by drugs, resulting in a drug–drug interaction (DDI). In the context of statin pharmacokinetics, substantial evidence indicates that the presence of OATP1B1 inhibitors exerts a comparable impact on systemic exposure and tolerability to that of genetic alterations.^9^, ^10^, ^11^, ^12^

Nevertheless, when reduced OATP1B1 function due to genetic predisposition is combined with transporter inhibition by co‐medications, cumulative effects may arise, resulting in the phenomenon of phenoconversion—a mismatch between the genetically predicted phenotype and the actual *in vivo* transporter activity. This phenomenon is mediated by drug–drug–gene interactions (DDGIs).^13^ Assessing DDGIs in clinical practice remains challenging, as it requires integrating genetic and pharmacological information into medication reviews.^11^, ^14^

One approach to address the interplay of genetics and drug‐induced changes in activity of pharmacokinetic (PK) contributors is the application of phenotyping probes.^15^, ^16^ These probes are typically exogenous substrate drugs administered to assess the actual activity of specific enzymes or transporters. Alternatively, phenotyping based on endogenous molecules is gaining increasing attention as it circumvents additional drug administration. In the context of OATP1B1, both preclinical and clinical studies have proposed to assess DDIs by quantifying the endogenous biomarkers coproporphyrin I (CPI) and CPIII^17^, ^18^ which are intermediates of heme biosynthesis. As a substrate of OATP1B1,^19^ especially CPI can be applied to identify whether a compound interacts with the transporter *in vitro*.^20^, ^21^ Moreover, several studies in healthy volunteers demonstrated that OATP1B1 inhibition by rifampicin or cyclosporine leads to dose‐dependent increases in CPI levels,^22^ mirroring changes in blood levels of well‐known OATP1B1 substrate drugs. Collectively, these findings support the use of CPs, particularly CPI, as reliable biomarkers for assessing OATP1B1‐mediated DDIs.^18^, ^23^ In clinical drug development, the recommended approach for employing an endogenous marker, such as CPI, involves quantifying its plasma concentrations at baseline and at multiple time points following drug administration, enabling the determination of *C*_max_ and area under the curve (AUC) ratios as indicators of transporter‐mediated effects.^24^

Emerging evidence also indicates that single timepoint measurements may be sufficient to characterize OATP1B1 activity. Notably, Neuvonen *et al*. demonstrated that plasma CPI concentrations measured at a single timepoint were ~ 68% higher in individuals carrying reduced function *SLCO1B1* variants compared with noncarriers.^25^ Their findings suggest that CPI levels can reliably reflect *in vivo* OATP1B1 activity, irrespective of the underlying cause of functional impairment. It should be noted, however, that these observations were derived from blood samples collected under controlled conditions, specifically at defined timepoints in fasted, healthy volunteers.

Building on these findings, the present study aimed to evaluate whether CPI measurements can be applied to determine OATP1B1 activity in a real‐world clinical setting. To this end, we analyzed samples from patients who underwent a pharmacist‐led medication review with integrated pharmacogenetic testing following therapy failure (TF) or adverse drug reactions (ADRs).^14^, ^26^, ^27^ The CPI concentrations measured at single timepoints were assessed in relation to genotype‐predicted OATP1B1 activity while also accounting for the influence of potential DDIs.

## MATERIALS AND METHODS

### Study design

The data and biological samples analyzed in this sub‐study approved by the local ethics committee (EKZN‐ID: 2024–02493) were derived from the case series study “Pharmacogenetic Testing of Patients with Unwanted Adverse Drug Reactions (ADRs) or Therapy Failure (TF)” (EKNZ‐ID: 2019–01452; ClinicalTrials.gov NCT04154553). This study is ongoing, recruiting patients in Switzerland at two community pharmacies providing primary care services and two hospital pharmacies offering specialized secondary care. All participants provided written informed consent. Medical history, medication, including self‐medication, diagnoses, ADRs, TF, and other negative experiences with pharmacotherapy, were systematically documented during a pharmacist‐led PGx counseling. Each patient also provided a buccal swab and two blood samples (EDTA blood and serum). The detailed study procedures have been described previously.^14^, ^26^ Participants were considered for inclusion in the sub‐study if they had been enrolled between October 2019 and December 2024 and met the following eligibility criteria: ≥ 18 years old, informed consent for the further use of genetic and personal data and biological material, both EDTA blood and serum samples available, documented medication records, and completed Stratipharm® PGx panel testing. Patients with visibly hemolytic serum samples were excluded.

### Pharmacogenetic analysis of *SLCO1B1*

DNA isolated from buccal swabs and blood samples was applied for the genotyping of four polymorphisms within the *SLCO1B1* gene. The genotyping of the variants rs4149056, rs11045819, rs2306283 was conducted by the commercial provider humatrix AG (Pfungstadt, Germany) as part of the Stratipharm® PGx panel testing as described elsewhere.^28^ The rs34671512 was genotyped in house. Briefly, genomic DNA was isolated from 250 μL of EDTA blood using the QIAmp DNA Blood Mini kit and the QIAcube (Quiagen, Hilden, Germany) following the manufacturer's instructions. Extracted DNA samples were analyzed for quantity and quality by spectrometry using the TECAN Infinite Pro 200 (Tecan, Männedorf, Switzerland). Subsequently, the genomic DNA was subjected to genotyping applying the TaqMan Genotyping chemistry (Applied Biosystems, Thermo Fisher Scientific, Reinach, Switzerland). Each 10 μL PCR reaction mix contained 10 ng of DNA, 5 μL of the TaqMan Genotyping Master Mix, and 0.5 μL of the primer/probe‐mix C__25605954_10. The PCR reaction was conducted using the QuantStudio 5 Real‐Time PCR System operated with QuantStudio Design and Analysis Software v1.5.3 (Applied Biosystems). For each patient, information on the polymorphisms rs4149056, rs11045819, rs2306283, and rs34671512 was used to determine the individual OATP1B1 alleles, and predict the corresponding haplotype. The haplotypes were then translated into the genotype‐predicted phenotypes as previously described^8^ (detailed information in **Table** **S1**).

### Quantification of CPs in clinical samples

CPI and CPIII were quantified in serum using a validated UPLC–MS/MS method adapted from Kinzi *et al*.^29^ Calibration curves were established over a concentration range of 0.25–20 ng/mL using standards prepared in a surrogate matrix (4% BSA (Sigma‐Aldrich, Buchs, Switzerland) in PBS (Thermo Fisher Scientific)), demonstrating excellent linearity (*r* ^2^ = 0.9994). The lower limit of quantification (LLOQ) was 0.25 ng/mL for CPI. Inter‐run accuracy ranged from 100% to 103%, and precision ranged from 3.7% to 8.3% across all quality control levels, meeting acceptance criteria (±15%) according to regulatory guidelines (FDA and EMA,^30^, ^31^). Briefly, calibration standards and quality control samples (LQC: 0.75 ng/mL; MQC: 8.0 ng/mL; HQC: 16 ng/mL) were prepared from stock solutions of CPI and CPIII (10 mg/mL in DMSO) diluted in acetonitrile/methanol/formic acid (v:v:v; 47.5:47.5:5). For sample preparation, 200 μL of serum were mixed with 200 μL of 1.25% ammonium hydroxide containing the internal standards [^15^N_4_]‐CPI and [^15^N_4_]‐CPIII at a concentration of 1 ng/mL each. Samples, calibration standards, and QCs were processed using Oasis MAX 96‐well μElution plates (30 μm; Waters) on a positive pressure manifold. Plates were conditioned with methanol and water, samples were loaded, and columns were washed with 1.25% ammonium hydroxide and methanol. Elution was performed twice with acetonitrile/methanol/formic acid (v:v:v; 47.5:47.5:5). Eluates were then dried under nitrogen at 50°C, reconstituted in 100 μL of the same solvent, and 20 μL injected for analysis. Chromatographic separation was achieved on an ACQUITY UPLC BEH C18 column (1.7 μm, 2.1 × 100 mm; Waters, Baden, Switzerland) coupled to an Agilent 6460 triple quadrupole mass spectrometer with AJS ESI (Agilent Technologies, Basel, Switzerland). Multiple reaction monitoring in positive mode monitored the transitions 655.3 → 596.3 and 655.3 → 537.3 for CPs, and 659.6 → 467.2 as well as 659.6 → 408.9 for the internal standards.

### Transport experiments

Cellular transport experiments were conducted as previously described.^20^ Briefly, MDCKII‐OATP1B1 cells were cultured in DMEM (Sigma‐Aldrich) supplemented with 10% FCS (BioConcept, Allschwil, Switzerland), 2 mM L‐glutamine (BioConcept), and 500 μg/mL Geneticin (Carl Roth, Arlesheim, Switzerland). For experiments, cells were seeded at a density of 75,000 cells per well in 24‐well plates. The following day, cells were stimulated with 2 mM sodium butyrate. The next day, cells were first washed with PBS and then equilibrated for 10 min in HBSS‐H consisting of Hanks' balanced salt solution (HBSS; Sigma‐Aldrich) supplemented with 10 mM HEPES (BioConcept). Transport was initiated by replacing the supernatant with HBSS‐H containing 1 μM CPI supplemented with solvent control (DMSO; Huberlab AG, Aesch, Switzerland), metformin (0.01–100 μM, Sigma‐Aldrich), atorvastatin (0.01–100 μM, Sigma‐Aldrich), semaglutide (0.005–10 μM, LubioScience, Zurich, Switzerland), or rosuvastatin as positive control (50 μM, Sigma‐Aldrich). After 10 min of incubation at 37°C in the dark, cells were washed twice with ice‐cold PBS and lysed in 1 mM HEPES with 1% Triton X‐100 (pH 6.5) for 30 min at 37°C. Protein content was determined from a 10 μL lysate aliquot using the Pierce BCA Protein Assay Kit (Thermo Fisher Scientific). In the remaining lysate, intracellular CPI levels were quantified after centrifugation (540 × *g*, 10 min, 4 °C) using fluorescence detection on a TECAN M200Pro plate reader (λ_ex 395 nm, λ_em 620 nm). Concentrations were calculated by linear regression against calibration standards (0.333–1,000 nM) prepared in the lysis buffer.

### Statistical analysis

Study data were collected and managed using REDCap, an electronic data capture tool, hosted at the University of Basel.^32^ Characterization of a medication interacting with OATP1B1 as a competitive or noncompetitive inhibitor was previously performed considering both *in vitro* and *in vivo* findings.^11^, ^33^ The list of all drugs exhibiting OATP1B1 inhibitory effects (referred to as putative OATP1B1 inhibitors) was used in the herein reported analyses. Data analysis was conducted using Microsoft Excel and GraphPad Prism (Version 10.2.0, San Diego CA, USA). Transport experiments were statistically analyzed using an Ordinary one‐way ANOVA with Dunnett's multiple comparisons test. To statistically compare CPI levels between patients not taking an inhibitor to patients that take a putative OATP1B1 inhibitor, a Mann–Whitney test was used after conducting a Shapiro–Wilk test to check for normal distribution. To compare CP levels between different genotype‐predicted phenotypes with or without putative inhibitor intake, a Kruskal–Wallis test with Dunn's multiple comparisons test was applied. The corresponding statistical test and the *P*‐value are stated within the context of the results.

## RESULTS

### Determination of CP levels in serum samples of patients

**Figure 1:**
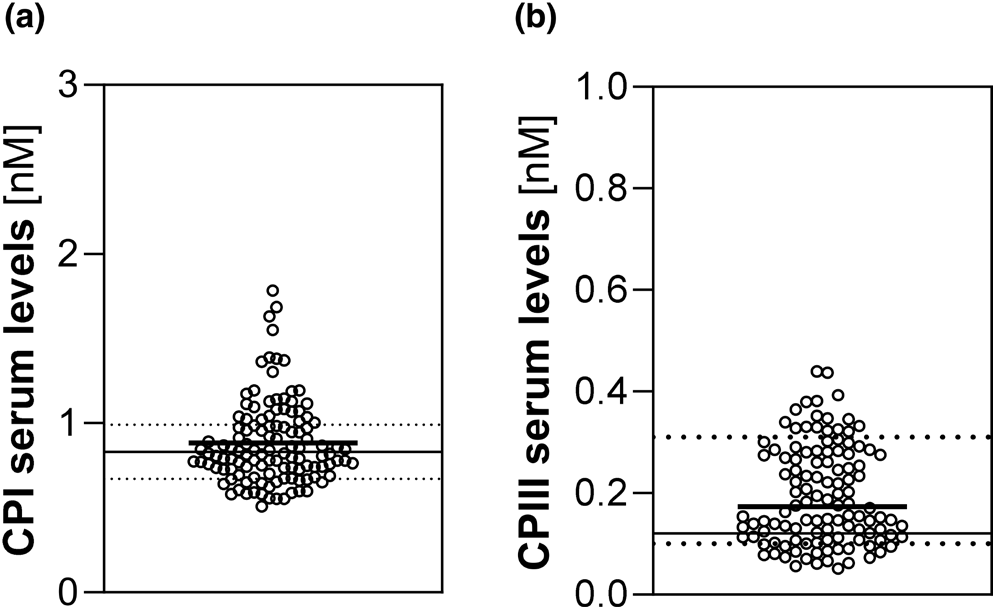
Serum CP concentrations in patients from the study cohort. A total of 119 serum samples were analyzed and individual CP values are displayed as circles together with the mean (nM) as horizon bar. CPI (a) levels were available for 119 samples, the depicted reference range mean (95% CI) is 0.83 nM (0.67–0.99 nM). CPIII (b) levels were available for 116 samples, the depicted reference range is based on previously published data^29^ and corresponds to 0.12 nM (0.10–0.31 nM).

A total of 119 patients were included in this sub‐study with a median age of 54 years [IQR 41–64]. 72 patients were female (60.5%) and 47 were male (39.5%) (**Table** **1**). CPI was detected in all 119 measured serum samples (**Figure** **1** **a**) with a mean concentration of 0.88 nM (95% CI: 0.84–0.93 nM) and the majority of measured concentrations being close to previously reported CPI levels in healthy volunteers.^23^ When applying our recently established CPI reference range (mean CPI concentration 0.83 nM, 95% CI: 0.67–0.99 nM),^34^ 16% (*n* = 19) of patients not receiving an OATP1B1 modulating drug (*n* = 95) had CPI levels exceeding this range, with a mean concentration of 1.13 nM. CPIII could only be detected in 116 samples. Those samples had a mean concentration of 0.20 nM (95% CI: 0.18–0.21 nM) (**Figure** **1** **b**) and only 14 patients out of the 95 that did not receive a putative OATP1B1 inhibitor were above the herein applied reference range (0.12 nM, 95% CI: 0.10–0.31 nM^29^) with a mean CPIII concentration of 0.33 nM.

**Table 1:** Overview of study population characteristics of the 119 enrolled patients

Characteristics	Description	Values
Sex	Female	60.5%
	Male	39.5%
Age in years	Median [IQR], (minimum, maximum)	54 [41–64] (18, 88)
Reported number of substances	Median [IQR], (minimum, maximum)	6 [3–9] (1, 18)
Patients with polypharmacy	≥ 5 reported substances	64.7%
Patients with OATP1B1 inhibitors	≥ 1 reported inhibitor	20.2%
	≥ 2 reported inhibitors	8.40%
	≥ 3 reported inhibitors	0.840%

### Influence of putative OATP1B1 inhibitor intake on CPI levels within the study population

**Figure 2:**
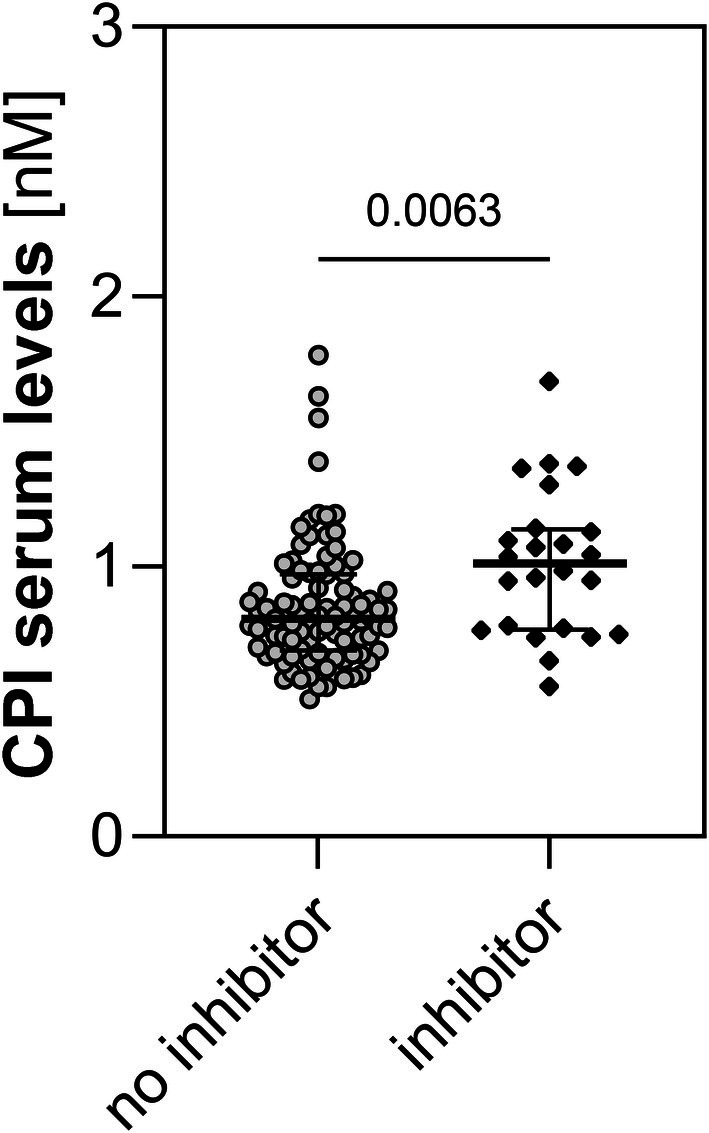
Effect of putative OATP1B1‐inhibitor intake on CPI levels in patients from our study cohort. Patients receiving a putative OATP1B1 inhibitor vs. those not taking such drug. Data are presented as median [IQR]; each point represents an individual patient. Group differences were evaluated using a Mann–Whitney test (*p* = 0.0063). Beforehand, data were tested for normal distribution using the Shapiro–Wilk test. Since the no inhibitor group did not meet the criteria for normal distribution (*p* < 0.0001), we assumed a non‐normal distribution for statistical testing.

At the time of the blood sampling, patients took a median of six substances [IQR 3–9], with more than half of the population (64.7%) classified as receiving polypharmacy, defined as the use of five or more substances. Within the study population, 24 patients (20.2%) were taking at least one putative OATP1B1 inhibitor (**Table** **1**). The definition of a putative OATP1B1 inhibitor in the analyses presented herein derives from previous data, where we extracted information on the interaction (*in vitro* and/or *in vivo*) with the transporter from the Certara Drug Interaction Database (DIDB) and Swissmedic (Swiss pharmacovigilance data).^11^, ^33^ The comparison between CPI levels in patients without the intake of a putative inhibitor and CPI levels in patients receiving at least one drug that exhibits OATP1B1 inhibitory potency, as shown in **Figure** **2**, revealed significantly higher CPI levels in patients with at least one putative OATP1B1 inhibitor (median CPI levels with interquartile range [IQR] in nM; no inhibitor vs. inhibitor; 0.81 [0.69–0.97] vs. 1.01 [0.77–1.14], Mann–Whitney test; *p* = 0.0063).

### Analysis of CPI levels correlated to the genotype‐predicted OATP1B1 phenotypes

**Figure 3:**
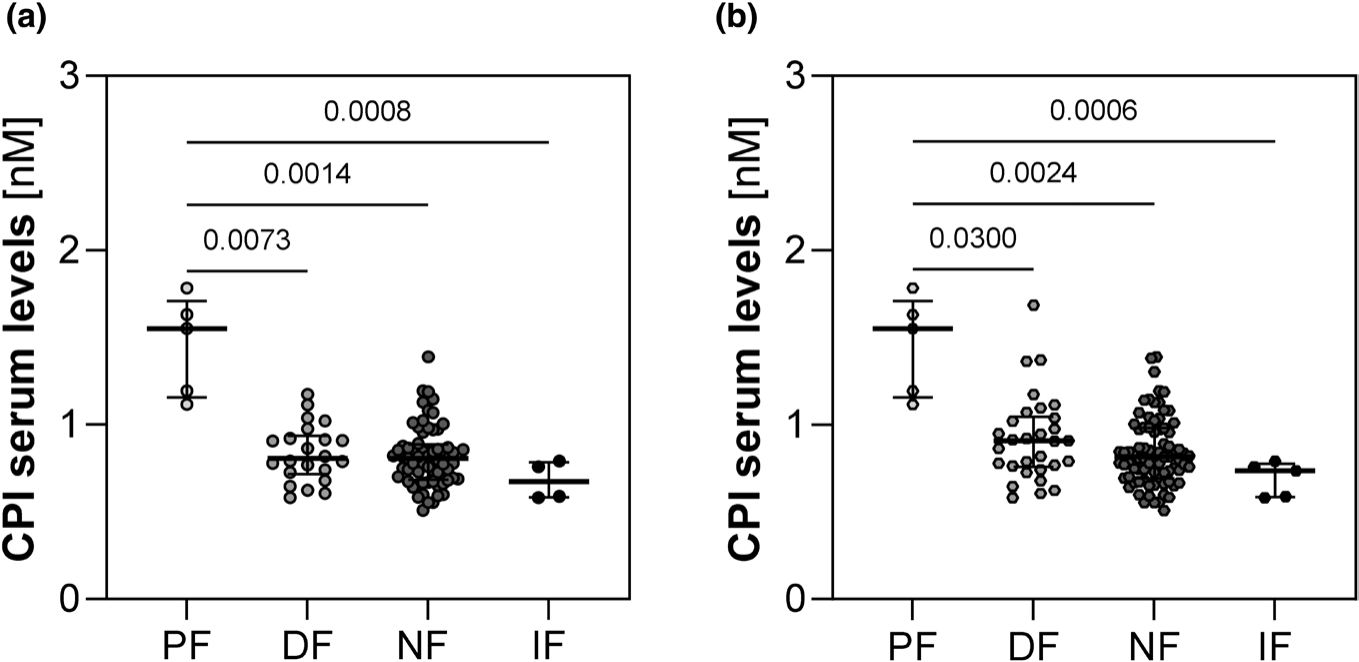
Effect of genotype‐predicted phenotypes on CPI concentrations. Among patients not taking a putative OATP1B1 inhibitor (a), CPI levels were compared across genotype‐predicted phenotypes. Data are presented as median [IQR]; each point represents an individual patient. Group differences were evaluated using a Kruskal–Wallis test with Dunn's multiple comparisons test. CPI levels from the full cohort of 119 patients (b) were similarly evaluated by genotype‐predicted phenotypes using a Kruskal–Wallis test with Dunn's multiple comparisons test: PF: poor function, DF: decreased function, NF: normal function, IF: increased function.

Based on the four *SLCO1B1* SNPs analyzed, we identified a haplotype distribution that differed significantly from the expected distribution in the European reference population^35^ (Chi‐square test; *p* = 2.83 × 10^−7^). However, no deviation was observed when comparing the observed and expected phenotype distributions in our and the European reference population (Chi‐square test; *p* = 0.763), see **Table** **S2** for details. Based on the OATP1B1 genotype‐predicted phenotypes, we intended to analyze the correlation between those and CPI levels. We first restricted our analysis to patients without the intake of a putative OATP1B1 inhibitor (**Figure** **3** **a**). Comparison of CPI levels between the different genotype‐predicted phenotype groups showed significantly higher median CPI levels in carriers of the OATP1B1 poor‐function phenotype (PF, median [IQR] in nM: 1.55 [1.16–1.71]) compared with the decreased (DF, median [IQR] in nM: 0.81 [0.72–0.94]; *p* = 0.0073), normal (NF, median [IQR] in nM: 0.81 [0.68–0.89]; *p* = 0.0014) and increased function (IF, median [IQR] in nM: 0.67 [0.58–0.78]; *p* = 0.0008, Kruskal–Wallis test with Dunn's multiple comparisons test) phenotypes. When analyzing the median CPI levels of all patients, irrespective of whether they were treated with at least one drug that has OATP1B1 inhibitory potential or not, similar results were obtained (**Figure** **3** **b**).

### Influence of drugs with potential effects on OATP1B1 activity on CPI levels in patients carrying the genotype‐predicted DF, NF, or IF phenotype

**Figure 4:**
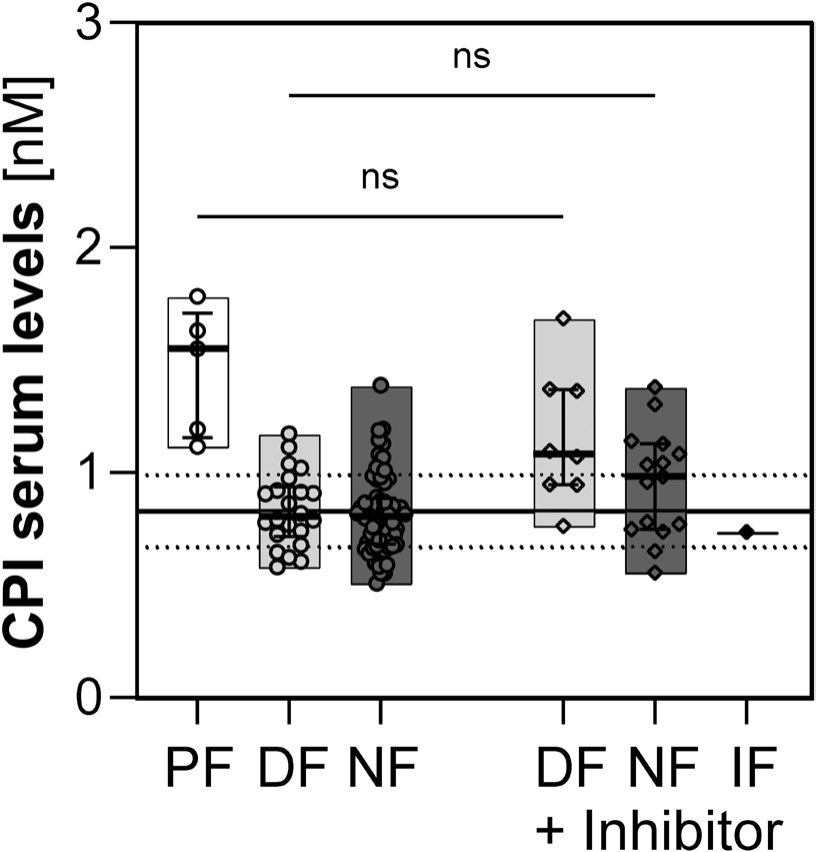
Comparison of CPI levels between patients carrying the PF, DF, or NF phenotype and not receiving putative OATP1B1 inhibitors and DF, NF, or IF phenotype patients receiving putative inhibitors, respectively. CPI serum levels in patients with the genotype‐predicted PF, DF, or NF phenotype who were not taking a potential OATP1B1 modulating drug were compared with levels in patients with genotype‐predicted DF, NF, or IF function phenotypes who were taking such a drug. Each point represents an individual patient; bars indicate the median with the IQR and boxes depict the min–max range. The reference range (mean with 95% CI) is shown (0.83 nM; 0.67–0.99 nM). Statistical significance was evaluated using a Kruskal–Wallis test with Dunn's multiple comparisons test.

In a next step, we compared the CPI levels in patients receiving putative OATP1B1 inhibitors and who carry the genotype‐predicted DF, NF, or IF phenotype to CPI levels in patients not receiving an inhibitory drug but carrying the PF, DF, or NF phenotype, respectively (**Figure** **4**, number of subjects per subgroup **Table** **S3**). Statistical analysis revealed no significant differences between the compared groups (median [IQR] in nM; PF vs. DF & Inh, DF vs. NF & Inh; 1.55 [1.16–1.71] vs. 1.08 [0.95–1.37], 0.81 [0.72–0.94] vs. 0.98 [0.75–1.13]; Kruskal–Wallis test with Dunn's multiple comparisons test, *p* > 0.05), suggesting that reduced function irrespective of its origin (genetic or inhibition) results in comparable high CPI levels in samples collected in this cohort. Additionally, comparison of patients in the NF group that did not receive an inhibitor and patients carrying the same genotype‐predicted phenotype that received a putative inhibitor revealed no significant difference (median [IQR] in nM; NF vs. NF & Inh; 0.81 [0.68–0.89] vs. 0.98 [0.75–1.13]; Kruskal–Wallis test with Dunn's multiple comparisons test, *p* > 0.05).

### Influence of atorvastatin, ezetimibe, semaglutide, or metformin intake on CP levels in different genotype‐predicted phenotype groups

**Figure 5:**
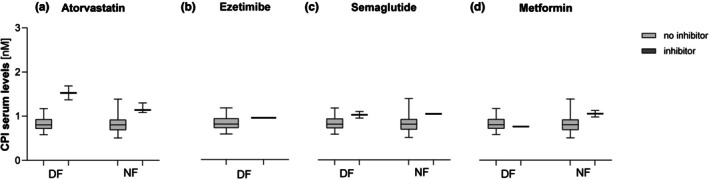
Effect of intake of atorvastatin (a), ezetimibe (b), semaglutide (c), and metformin (d) on CPI levels within different genotype‐predicted phenotype groups. Data are presented as median with the range (min–max).

The four most frequently prescribed drugs with a potential impact on OATP1B1 in our study population (*n* = 119) were atorvastatin (*n* = 11, 9.2%), metformin (*n* = 10, 8.4%), semaglutide (*n* = 8, 6.7%), and ezetimibe (*n* = 4, 3.4%). Descriptive analysis suggests that patients receiving atorvastatin (**Figure** **5** **a**) exhibited numerically higher CPI levels than those not taking the drug, irrespective of their genotype‐predicted phenotypes. Similar observations were made for patients treated with ezetimibe (**Figure** **5** **b**) or semaglutide (**Figure** **5** **c**), where CPI levels appeared numerically higher compared with non‐users. For metformin (**Figure** **5** **d**), patients classified within the NF phenotype and receiving metformin appeared to have numerically higher CPI levels compared with those without metformin intake. In contrast, within the DF phenotype group, the CPI level of the single patient receiving metformin appeared comparable to that of patients not taking the putative inhibitor. Similar observations were made for CPIII levels, although with less pronounced numerical differences (**Figure** **S1**).

### Interaction of atorvastatin, semaglutide, and metformin with the OATP1B1‐mediated uptake of CPI

**Figure 6:**
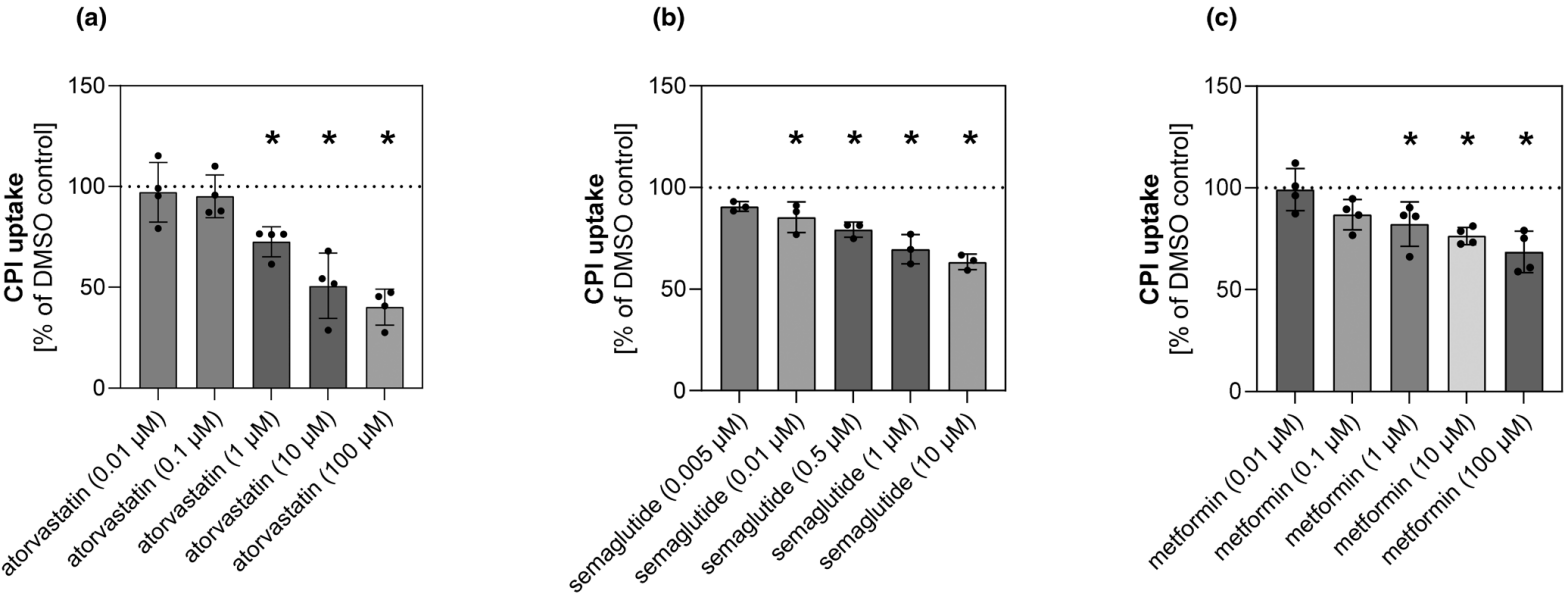
Effect of atorvastatin (a), semaglutide (b), and metformin (c) on the OATP1B1‐mediated CPI uptake. The impact of each drug on CPI uptake was assessed in MDCKII‐OATP1B1 cells. Data are presented as mean ± SD as % of solvent control with individual data points shown as dots from *n* = 4 independent experiments (for atorvastatin and metformin) and *n* = 3 independent experiments for semaglutide, each performed in technical triplicates. Statistical significance was evaluated using an Ordinary one‐way ANOVA with Dunnett's multiple comparisons test (*p < 0.05).

In a last step, we performed an *in vitro* transport experiment to address the influence of atorvastatin, semaglutide, and metformin on the OATP1B1‐mediated uptake of CPI. As shown in **Figure** **6** **a**, the presence of 1 μM, 10 μM, and 100 μM atorvastatin significantly reduced the OATP1B1‐mediated cellular uptake of CPI compared with solvent control, while no such effect was observed for the concentrations 0.01 μM and 0.1 μM. Similar results were obtained when testing the interaction of semaglutide (**Figure** **6** **b**) with the OATP1B1‐mediated uptake of CPI, where we observed a significant reduction of the CPI uptake in the presence of 0.01 μM, 0.5 μM, 1 μM, and 10 μM semaglutide. Lastly, a similar pattern was observed when assessing the influence of metformin on the OATP1B1‐mediated CPI uptake. Analysis revealed a significant reduction of CPI uptake when cells were treated with 1 μM, 10 μM, and 100 μM metformin, while no such effect was observed for metformin at concentrations of 0.01 μM and 0.1 μM (**Figure** **6** **c**).

## DISCUSSION

In this study, we utilized samples from the case series “Pharmacogenetic Testing of Patients with Unwanted Adverse Drug Reactions (ADRs) or Therapy Failure (TF)” to evaluate the utility of CPI measurement as an indicator of OATP1B1 activity in clinical routine. As these analyses were based on a subset of patients recruited in this ongoing case series study without stratification for sex, the resulting imbalance reflects the underlying study population and should be considered when interpreting the findings. Most evidence supporting CPI as an endogenous biomarker for predicting OATP1B1 activity comes from controlled clinical studies, either pairing volunteer genotyping with fasting CPI measurements^25^ or assessing CPI pharmacokinetics before and after dosing with known OATP1B1 inhibitors using pre‐ and post‐dose blood sampling.^36^, ^37^, ^38^ However, this controlled sampling is mostly not feasible in clinical practice. To address this limitation, we quantified CP levels in single timepoint serum samples from patients who underwent pharmacist‐led medication reviews with integrated pharmacogenetic testing following TF or ADRs, and combined these data with a detailed assessment of their medication records and OATP1B1 genotype‐predicted phenotypes. To contextualize observed CPI concentrations, we compared them to previously reported CPI levels in healthy adults^23^ as well as to a mean reference CPI level of 0.83 nM (95% CI: 0.67–0.99 nM) derived from a previously conducted literature review and following meta‐analysis that included reported CPI levels of healthy adults.^34^ This comparison revealed mean CPI levels comparable to published values with about 25% of all subjects exhibiting levels higher than the reference range. About 64.7% of the total population were classified as receiving polypharmacy. As widely reported in the literature, polypharmacy is a well‐established contributor to both ADR and TF.^39^, ^40^ This is particularly relevant in patients with cardiovascular diseases, where statins are frequently prescribed,^41^ a drug class that accounts for ~93% of all lipid lowering therapies.^42^ Among these, atorvastatin is the most frequently used statin in Switzerland^43^ and has also been identified as the most commonly prescribed statin across Europe.^44^ Summarized by Stäuble *et al*.^11^, ^33^ and confirmed by our *in vitro* data, atorvastatin interacts with OATP1B1. In our study population, just over 20% of patients were taking at least one putative OATP1B1 inhibitor, with atorvastatin, metformin, semaglutide, and ezetimibe being the most frequently used ones. Those drugs were selected based on previously published data suggesting interaction with OATP1B1 *in vitro* and/or *in vivo*.^11^, ^33^ Furthermore, we considered their frequency of use in clinics with the aim of reflecting real‐world prescription patterns.^33^ Our *in vitro* results revealed differential interaction potential, with atorvastatin exerting the strongest effect on the OATP1B1‐mediated CPI transport, followed by semaglutide and metformin. These findings align with previously published *in vitro* data, which similarly identified atorvastatin^45^, ^46^ as an *in vitro* OATP1B1 inhibitor, and clinical data indicating the inhibitory potential of semaglutide,^47^ and metformin.^48^ For ezetimibe, earlier studies^20^ demonstrated that its active metabolite, ezetimibe–glucuronide, exhibits inhibitory activity toward OATP1B1. An important consideration when interpreting these data is the discrepancy between the classification of the investigated drugs as putative OATP1B1 inhibitors in this study and the availability of clinical evidence. While *in vitro* data show their inhibitory potential, *in vivo* findings often indicate only modest effects. This highlights a limitation in the translation of *in vitro* findings to *in vivo* settings. In this context, it is important to consider not only *in vitro* potency (e.g., IC_50_ or K_i_), but also the systemic and hepatic drug concentrations achieved at therapeutic dosing. Even when exposure–potency ratios exceed commonly used thresholds, false‐positive predictions may occur, and clinical DDI studies should therefore be considered more informative. When comparing CPI levels between the ~20% of patients receiving a putative OATP1B1 inhibitor and those not exposed to drugs classified as OATP1B1 inhibitors according to Stäuble *et al*.,^11^ we observed a significant increase in CPI concentrations among inhibitor users. This finding indicates that CPI could serve as a marker to detect OATP1B1 inhibition in a multimorbid, polymedicated clinical cohort. This is further supported by our drug‐specific analysis, which indicated that CPI levels appeared numerically higher in patients treated with each of the four most common inhibitory drugs—independent of their genotype‐predicted phenotype. The visual magnitude of CPI elevation appeared highest with atorvastatin, followed by ezetimibe, semaglutide, and metformin, consistent with the inhibitory potency observed in our *in vitro* experiments. Since this sub‐analysis reduced the number of patients per group, the results are exploratory and should be interpreted with caution.

Despite that, our observations align with previously published reports on the effects of OATP1B1 inhibitors on CPI levels.^17^, ^20^, ^22^, ^38^ Additionally, considering the CPI levels in different genotype‐predicted OATP1B1 phenotypes, we observed significantly higher CPI levels in the PF group compared with the DF, NF, and IF group in line with previously published studies in healthy volunteers that reported significant differences between *SLCO1B1* phenotype groups^25^ or in carriers of the c.521 T>C variant.^49^ However, there was a substantial overlap in CPI levels in patients of different genotype‐predicted phenotypes. Considering that the patients in our cohort were experiencing ADRs or TF, and as such clinical conditions may produce inflammatory responses, hepatic or renal toxicity, or metabolic disturbances thereby altering organ function, they may introduce greater variability in CPI values, potentially obscuring phenotype‐related differences between groups other than the PF group.

By integrating both genetic and inhibitory‐related data, we identified no significant difference in CPI levels between patients with a PF phenotype who were not receiving an OATP1B1 inhibitory drug and patients with a DF phenotype who received such drugs. A similar pattern was observed in additional comparisons, including patients with a DF phenotype without the intake of inhibitory drugs vs. those with a NF phenotype receiving a putative inhibitor, as well as patients with a NF phenotype without inhibitory medication compared with a patient with an IF phenotype receiving an inhibitory drug. These findings suggest that diminished OATP1B1 activity, whether caused by genetic variation or drug‐mediated inhibition, results in similarly elevated CPI levels in this clinical cohort, providing first evidence that CPI could be used to identify drug–drug–gene interactions. This becomes particularly apparent when examining individual patients as examples. In multimorbid and polymedicated patients, such as those in our cohort, inferring the functional OATP1B1 phenotype directly from genotype can be challenging. Introducing an activity score‐based approach, analogous to the established CYP2D6 activity score system,^50^ may therefore help to simplify genotype interpretation and improve phenotype prediction. Because no activity score model currently exists for transporters, our proposed scoring concept is informed by activity score frameworks used for other proteins as described before.^50^ To illustrate the challenge of interpretation, we want to highlight three patients: Patient 1 is not taking a putative OATP1B1 inhibitor and carries the genotype‐predicted PF phenotype (activity score = 0). In our analysis, Patient 1 had a measured CPI level of 1.63 nM. Patient 2 has a genotype‐predicted DF phenotype (activity score = 1). In this patient, we measured a CPI level of 1.04 nM. Patient 3 had the same DF phenotype (activity score = 1). When determining CPI in this patient, we observed a level of 1.69 nM. Considering only the genotype‐predicted phenotypes in our analysis, these CPI values do not align consistently within Patient 2 and Patient 3, as both were assigned a DF phenotype. Incorporating information on medication intake further clarifies this example. For Patient 3, we identified the use of atorvastatin as a drug that exhibits OATP1B1 inhibitory potential. Taking into account the different inhibitory potency observed in our *in vitro* experiments, we assumed different reductions of transporter activity and assigned atorvastatin an activity score deduction of −1 (potent inhibitor). Adjusting the genotype‐based activity score of 1 accordingly yielded a revised activity score of 0. This adjusted score, which is associated with a PF phenotype, aligns with the measured CPI level of 1.69 nM, reflecting a phenoconversion from the DF to a PF‐like functional state.

While the focus of our analyses was on the about 20% of patients treated with an OATP1B1 inhibitory drug, it is noteworthy that about 16% of the remaining 80% that were not exposed to such drugs, also exhibited CPI levels above previously reported concentrations in healthy volunteers.^23^, ^34^ This could be attributed to the high prevalence of patients with the PF phenotype within this group. In addition, the CPI values we refer to were derived from clinical studies conducted exclusively in young, healthy volunteers, whereas our study population consisted of patients with ADRs or TF, often with multimorbidity and polymedication. These differences may contribute to higher and more variable CPI levels observed in our cohort. Furthermore, the relatively small sample size, particularly in subgroup analyses, represents an important limitation, and the findings should therefore be considered exploratory and interpreted with caution.

Taken together, our findings provide initial evidence that CPs, particularly CPI, may serve as biomarkers for detecting drug–drug–gene interactions affecting OATP1B1 function. However, further investigations in larger and more diverse patient populations, including older individuals and those with comorbidities, are needed to better understand factors influencing CPI levels and to assess their suitability in real‐world clinical settings to support safer and more effective drug therapy.

## CONFLICTS OF INTEREST

The authors declared no competing interests for this work.

## FUNDING

No funding was received for this work.

## AUTHOR CONTRIBUTIONS

L.P., A.Z., C.S., and H.M.z.S. wrote the manuscript; L.P., C.S., and H.M.z.S. designed the research; L.P., A.Z., A.B., C.J., K.E.H., F.W., M.L.L., and S.A. performed the research; L.P., A.Z., C.S., and H.M.z.S. analyzed the data.

## Supporting information


Data S1.
