# Supplementary material for: Biomarker‐Based Prediction of OATP1B1 Activity in Clinical Routine—Investigating Coproporphyrins as Markers for Drug–Drug–Gene Interactions
Source: Clin Pharmacol Ther. 2026 May 18;120(2):492–500. doi: 10.1002/cpt.70335 (PMC13339200; doi:10.1002/cpt.70335)
Supplement: Supplementary file 1 — Data S1. [file CPT-120-492-s001.pptx]

## Slide 1
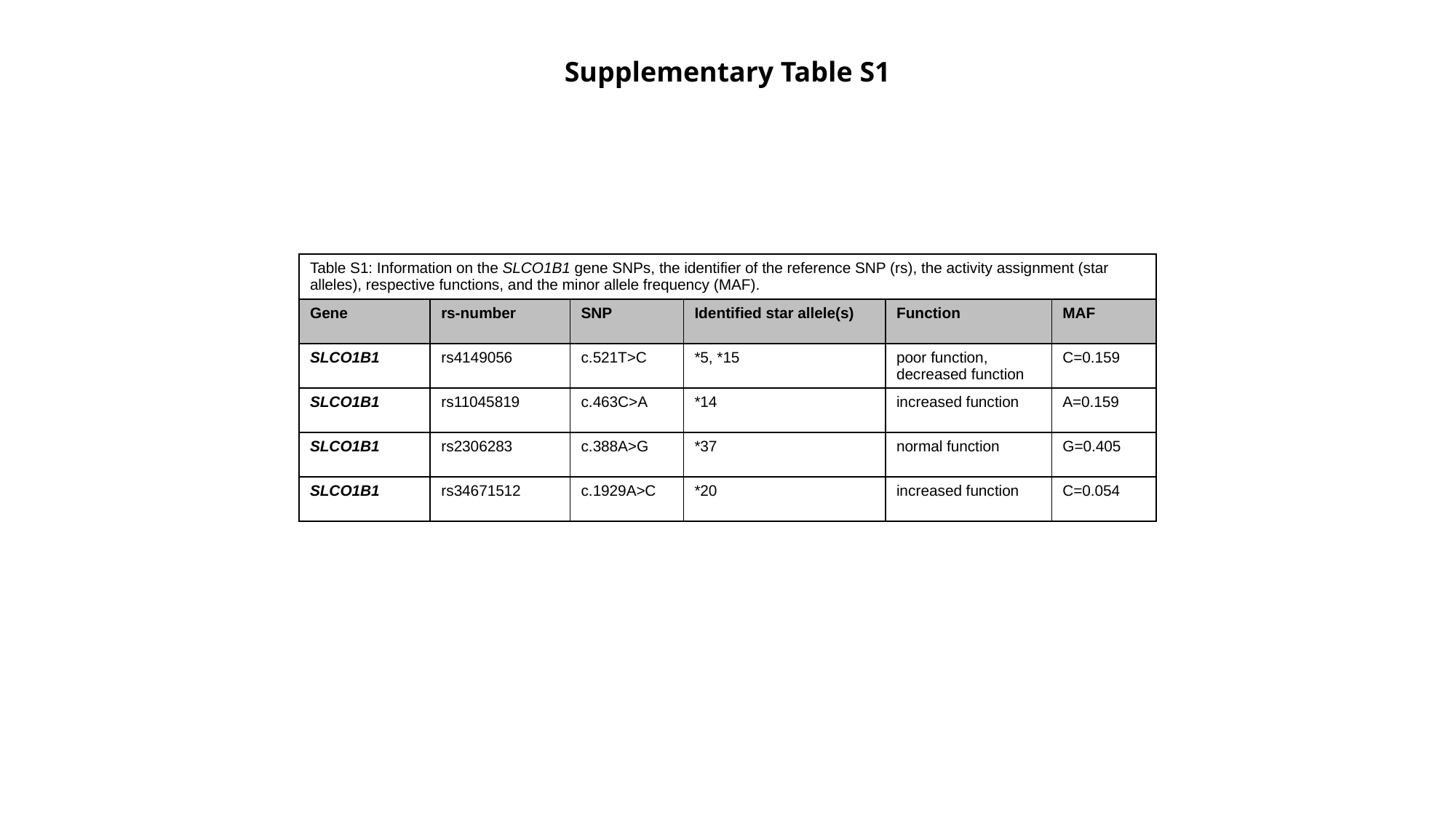

Supplementary Table S1
| Table S1: Information on the SLCO1B1 gene SNPs, the identifier of the reference SNP (rs), the activity assignment (star alleles), respective functions, and the minor allele frequency (MAF). | | | | | |
| --- | --- | --- | --- | --- | --- |
| Gene | rs-number | SNP | Identified star allele(s) | Function | MAF |
| SLCO1B1 | rs4149056 | c.521T>C | \*5, \*15 | poor function, decreased function | C=0.159 |
| SLCO1B1 | rs11045819 | c.463C>A | \*14 | increased function | A=0.159 |
| SLCO1B1 | rs2306283 | c.388A>G | \*37 | normal function | G=0.405 |
| SLCO1B1 | rs34671512 | c.1929A>C | \*20 | increased function | C=0.054 |

## Slide 2
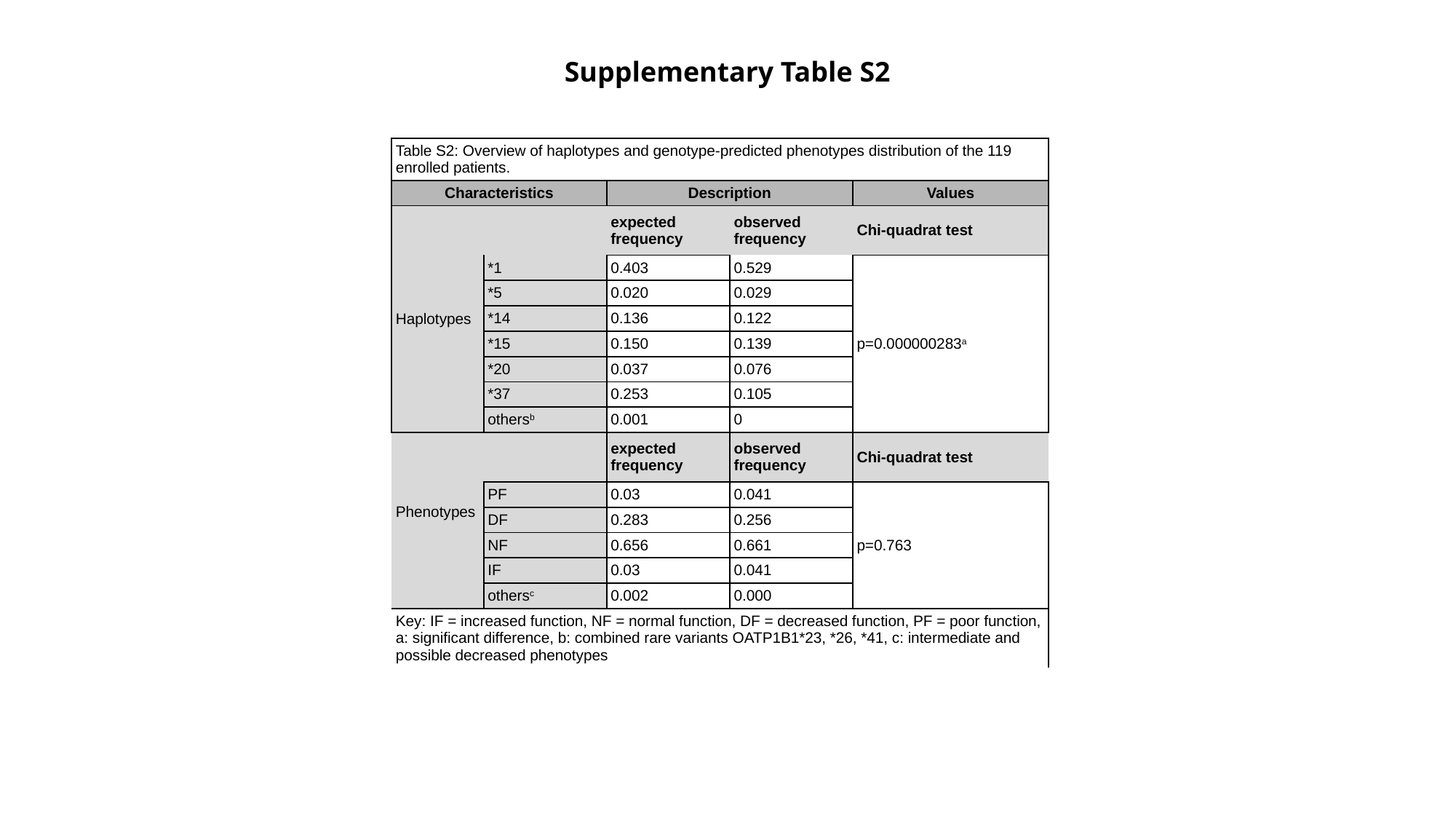

Supplementary Table S2
| Table S2: Overview of haplotypes and genotype-predicted phenotypes distribution of the 119 enrolled patients. | | | | |
| --- | --- | --- | --- | --- |
| Characteristics | | Description | | Values |
| Haplotypes | | expected frequency | observed frequency | Chi-quadrat test |
| | \*1 | 0.403 | 0.529 | p=0.000000283a |
| | \*5 | 0.020 | 0.029 | |
| | \*14 | 0.136 | 0.122 | |
| | \*15 | 0.150 | 0.139 | |
| | \*20 | 0.037 | 0.076 | |
| | \*37 | 0.253 | 0.105 | |
| | othersb | 0.001 | 0 | |
| Phenotypes | | expected frequency | observed frequency | Chi-quadrat test |
| | PF | 0.03 | 0.041 | p=0.763 |
| | DF | 0.283 | 0.256 | |
| | NF | 0.656 | 0.661 | |
| | IF | 0.03 | 0.041 | |
| | othersc | 0.002 | 0.000 | |
| Key: IF = increased function, NF = normal function, DF = decreased function, PF = poor function, a: significant difference, b: combined rare variants OATP1B1\*23, \*26, \*41, c: intermediate and possible decreased phenotypes | | | | |

## Slide 3
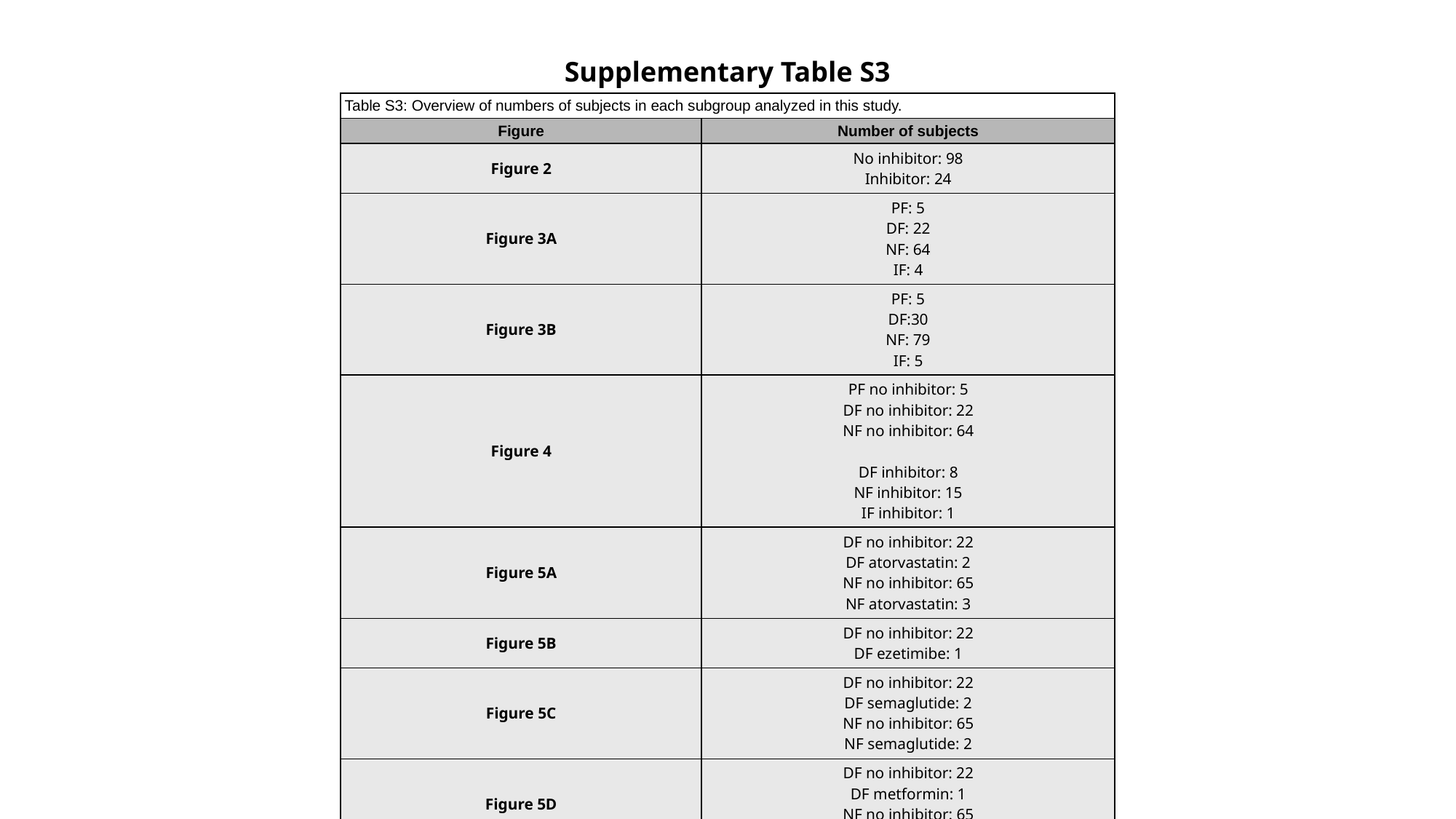

Supplementary Table S3
| Table S3: Overview of numbers of subjects in each subgroup analyzed in this study. | |
| --- | --- |
| Figure | Number of subjects |
| Figure 2 | No inhibitor: 98 Inhibitor: 24 |
| Figure 3A | PF: 5 DF: 22 NF: 64 IF: 4 |
| Figure 3B | PF: 5 DF:30 NF: 79 IF: 5 |
| Figure 4 | PF no inhibitor: 5 DF no inhibitor: 22 NF no inhibitor: 64 DF inhibitor: 8 NF inhibitor: 15 IF inhibitor: 1 |
| Figure 5A | DF no inhibitor: 22 DF atorvastatin: 2 NF no inhibitor: 65 NF atorvastatin: 3 |
| Figure 5B | DF no inhibitor: 22 DF ezetimibe: 1 |
| Figure 5C | DF no inhibitor: 22 DF semaglutide: 2 NF no inhibitor: 65 NF semaglutide: 2 |
| Figure 5D | DF no inhibitor: 22 DF metformin: 1 NF no inhibitor: 65 NF metformin: 2 |
| Key: IF = increased function, NF = normal function, DF = decreased function, PF = poor function | |

## Slide 4
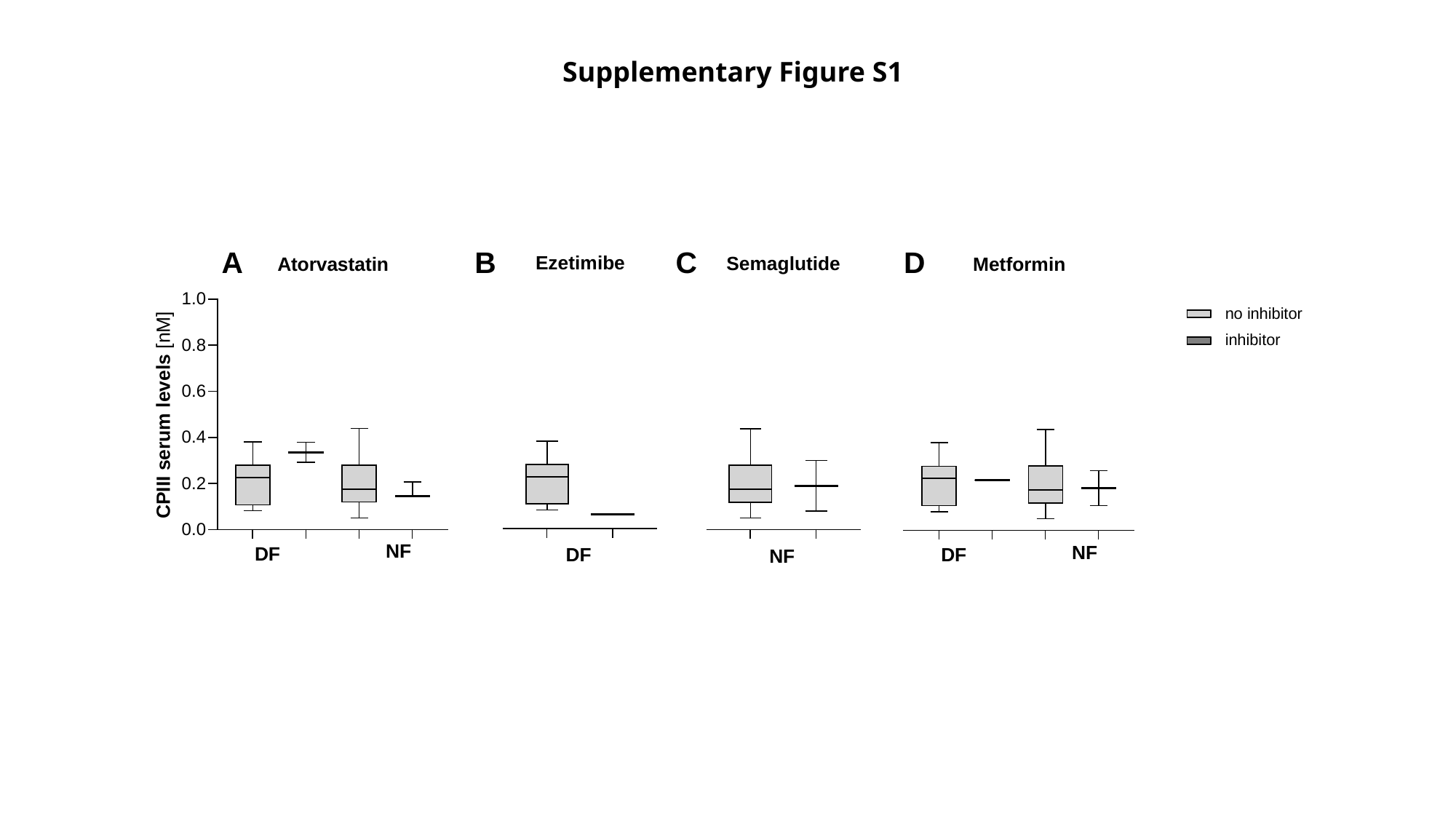

Supplementary Figure S1
